# 625. The Hidden Cost of Dalbavancin: OPAT-RN Time Spent on Coordination for Patients with Substance Use Disorder

**DOI:** 10.1093/ofid/ofab466.823

**Published:** 2021-12-04

**Authors:** Alyse Douglass, Heather Mayer, Kathleen Young, Amber C Streifel, Jina Makadia, James Lewis, Monica K Sikka

**Affiliations:** 1 Oregon Health and Sciences University, Portland, Oregon; 2 Oregon Health & Science University, Portland, OR; 3 Oregon Health and Science University, Portland, Oregon

## Abstract

**Background:**

The use of dalbavancin (DAL) enhances the management of serious gram-positive infections in people with substance use disorder (SUD) by eliminating the need for central lines, weekly lab monitoring, and may decrease length of hospitalizations. Though administered weekly, care coordination for DAL is often complex, due to variable access to resources, insurance variation and treatment settings. Our institution uses OPTIONS-DC, a multi-disciplinary discharge planning conference facilitated by an outpatient parenteral antimicrobial therapy (OPAT) registered nurse (RN) to determine safe treatment plans while emphasizing patient preference for hospitalized patients with SUD and serious infections. When DAL is selected for treatment, patients are enrolled in the RN-led OPAT program for coordination and monitoring. DAL has been shown to result in monetary savings but these estimates have yet to incorporate the true cost of coordination.

**Methods:**

We conducted a retrospective chart review of OPAT staff interventions required to coordinate DAL doses for patients with SUD (identified via ICD-10 code or chart notes). Additionally, we recorded in real time, the amount of time spent per intervention over a one month period for 7 additional patients.

**Results:**

53 courses of DAL in patients with SUD were included with a variety of dosing regimens and treatment settings (Table 1). 41 (77%) patients endorsed IV substance use. 68% of patients received DAL for osteomyelitis or endocarditis. The majority were insured by Oregon Medicaid (70%). The number of RN interventions per course averaged 3.35 with the most common being coordinating with patients and vendors (Table 2). The average time spent per patient course during a one-month sample was 39.4 minutes (range 15 – 58 minutes). The most time-consuming interventions were conducting the OPTIONS-DC conferences and attempting to reach patients after hospital discharge. Readmission for adverse effects or infection occurred for 4 (8%) patients.

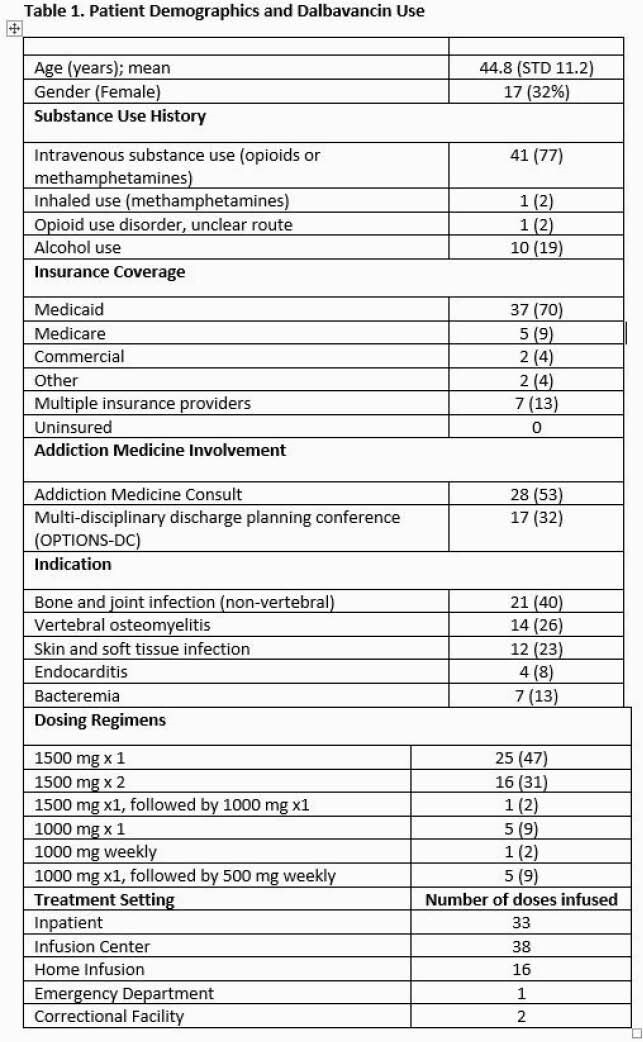

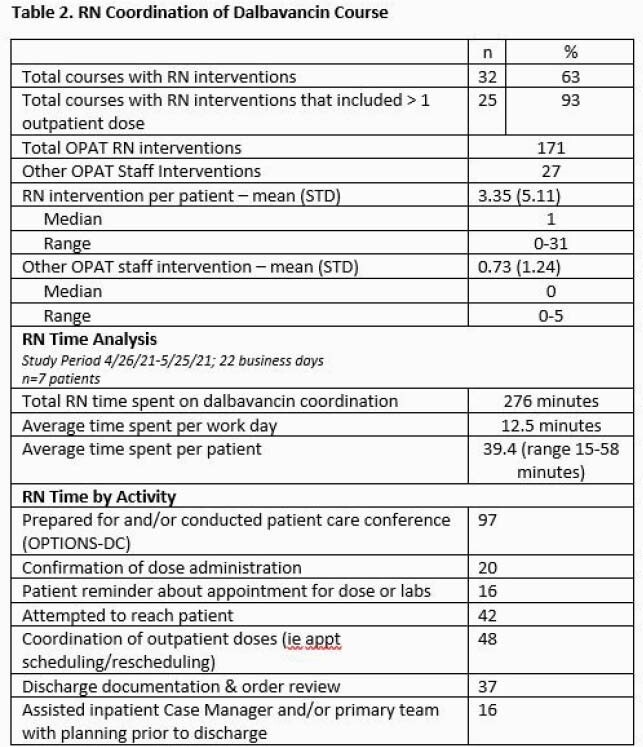

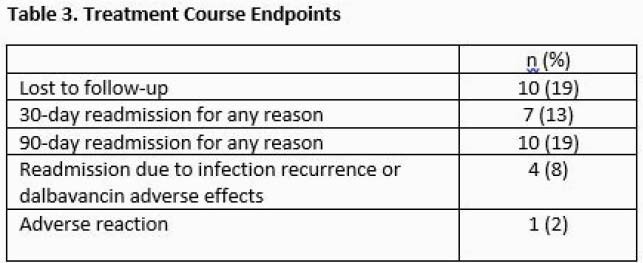

**Conclusion:**

The OPAT-RN time required to coordinate outpatient DAL for patients with SUD is substantial. This enhanced coordination allows for potential cost savings to health systems.

**Disclosures:**

**Amber C. Streifel, PharmD, BCPS**, **Melinta** (Advisor or Review Panel member) **Monica K. Sikka, MD**, **FG2** (Scientific Research Study Investigator)

